# Developing molecular-level models for organic field-effect transistors

**DOI:** 10.1093/nsr/nwaa167

**Published:** 2020-07-18

**Authors:** Haoyuan Li, Jean-Luc Brédas

**Affiliations:** School of Chemistry and Biochemistry, and Center for Organic Photonics and Electronics, Georgia Institute of Technology, Atlanta, GA 30332, USA; Department of Chemistry and Biochemistry, University of Arizona, Tucson, AZ 85721, USA; School of Chemistry and Biochemistry, and Center for Organic Photonics and Electronics, Georgia Institute of Technology, Atlanta, GA 30332, USA; Department of Chemistry and Biochemistry, University of Arizona, Tucson, AZ 85721, USA

**Keywords:** organic semiconductors, charge transport, kinetic Monte Carlo simulations, mobility measurements, gradual channel approximation

## Abstract

Organic field-effect transistors (OFETs) are not only functional devices but also represent an important tool for measuring the charge-transport properties of organic semiconductors (OSs). Thus, efforts to understand the performance and characteristics of OFET devices are not only useful in helping achieve higher device efficiencies but also critical to ensuring accuracy in the evaluations of OS charge mobilities. These studies rely on OFET device models, which connect the measured current characteristics to the properties of the OSs. Developing such OFET models requires good knowledge of the charge-transport processes in OSs. In device active layers, the OS thin films are either amorphous (e.g. in organic light-emitting diodes and organic solar cells) or crystalline (e.g. those optimized for charge transport in OFETs). When the electronic couplings between adjacent OS molecules or polymer chain segments are weak, the charge-transport mechanism is dominated by hopping processes, which is the context in which we frame the discussion in this Review. Factors such as disorder, mobility anisotropy, traps, grain boundaries or film morphology all impact charge transport. To take these features fully into account in an OFET device model requires considering a nano-scale, molecular-level resolution. Here, we discuss the recent development of such molecular-resolution OFET models based on a kinetic Monte Carlo approach relevant to the hopping regime. We also briefly describe the applicability of these models to high-mobility OFETs, where we underline the need to extend them to incorporate aspects related to charge delocalization.

## Teaser

Organic field-effect transistors are functional devices that have applications ranging from sensors to electrical circuits or data storage. They are also widely used in the characterization of the charge-carrier mobilities of organic semiconductors. This review summarizes recent advances in the development of theoretical models that can describe the operation of these devices at the molecular scale.

## INTRODUCTION

Organic field-effect transistors (OFETs) are functional devices that have applications ranging from sensors to electrical circuits or data storage [1,2]. They are also tools widely used in the characterization of the charge-carrier mobilities of organic semiconductors (OSs) [3,4]. Therefore, gaining a better understanding of the OFET device characteristics is a key step in the development of more efficient OSs and the extension of their applications. Device models are fundamental to the characterization of OFETs, as they provide the relationships between macroscopic observables such as current densities and microscopic, molecular-level features of the organic semiconductors. However, developing accurate and reliable OFET device models turns out to be challenging. There are many factors that affect charge-transport processes, such as disorder, mobility anisotropy, presence of traps and grain boundaries, and details of film morphology [5–7]; incorporating all of them simultaneously in an OFET model is not straightforward. Also, an accurate OFET model needs to take into account the microscopic charge-transport mechanism. Here, we will focus on instances where charge transport occurs mainly through a hopping mechanism, i.e. instances where the electronic couplings between adjacent OS molecules or polymer chain segments are weak.

To fully consider the microscopic features requires working with a molecular resolution. One of the common molecular-level methods is kinetic Monte Carlo (KMC), which has been successfully applied to study charge transport in organic semiconductors and their electronic devices [8–14]. Recently, molecular-level OFET models based on the KMC approach have been developed [15], which have allowed a description of fundamental OFET aspects that were difficult to comprehend earlier. They also proved useful in understanding some of the characteristics of high-mobility OFETs, even though delocalization aspects are still to be incorporated. Here, we summarize the latest progress made in the development of such molecular-level OFET models and their applications. This Review is structured as follows: we first briefly introduce the hopping transport mechanism in organic semiconductors; then, we describe the most widely used OFET device model; finally we proceed to a discussion of the KMC OFET models and their applications.

## CHARGE TRANSPORT IN ORGANIC SEMICONDUCTORS

In this section, we offer a brief introduction to charge transport in organic semiconductors, which is the basis of OFET operation, in the context of the hopping transport mechanism. For a more in-depth description, readers are referred to reviews devoted to this topic [2,5–7,16–23].

We recall that in crystalline inorganic semiconductors such as silicon, atoms are held together by covalent bonds that usually induce strong electronic couplings in three dimensions and charge delocalization. Charge carriers are then expected to move freely in the valence or conduction band until scattered by phonons or defects. This picture, however, does not hold true in many organic semiconductor thin films.

**Figure 1.**
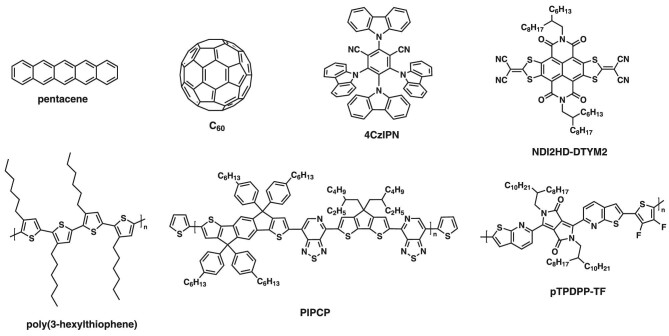
Chemical structures of representative organic semiconductors. Molecular semiconductors (top): pentacene [28], C_60_ [29,30], 4CzIPN (1,2,3,5-tetrakis(carbazol-9-yl)-4,6-dicyanobenzene) [31] and NDI2HD-DTYM2 (2,2'-[2,8-bis(2-hexyldecyl)-1,2,3,7,8,9-hexahydro-1,3,7,9-tetraoxo[1,3]benzodithiolo[4,5,6,7-*lmn*][1,3]dithiolo[4,5-*f*][3,8]phenanthroline-5,11-diylidene]bis[propanedinitrile]) [32,33]. Polymeric semiconductors (bottom): (polymeric semiconductors) poly(3-hexylthiophene) [34], PIPCP (synthesized via the copolymerization of 4,4'-(4,4-bis(2-ethylhexyl)-4*H*-cyclopenta[2,1-*b*:3,4-*b'*]dithiophene-2,6-diyl)bis(7-bromo-[1,2,5]thiadiazolo[3,4-*c*]pyridine) and (4,4,9,9-tetrakis(4-hexylphenyl)-4,9-dihydro-*s*-indaceno[1,2-*b*:5,6-*b'*]dithiophene-2,7-diyl)bis(trimethylstannane)) [35], and pTPDPP-TF (synthesized via the copolymerization of thieno[2,3-*b*]pyridine diketopyrrolopyrrole (TPDPP) and 3,4-difluorothiophene) [36].

Organic semiconductors are π-conjugated small molecules or polymer chains (see Fig. 1) held together by weak van der Waals interactions, which make them prone to disorder. In some cases, a band-like mechanism and a hopping mechanism can coexist, with one mechanism being prevalent as a function of factors such as molecular packing, electronic coupling and temperature; to ensure a band regime requires highly crystalline/molecularly aligned organic films and large intermolecular electronic couplings (dominating over electron-vibration couplings), which results in charge transport controlled by delocalization effects [24–27].

However, in many situations, the OS films are amorphous or have a mixed crystalline/amorphous nature, and are subject to a complex morphology. This is the case typically in the active layers of organic photovoltaic devices and organic light-emitting diodes as well as in many OFET layers, in which the electronic couplings between molecules or polymer chain segments are weak and charge carriers are mostly localized on individual molecules or chain segments. The hopping transport mechanism then dominates at room temperature, with charge carriers hopping from molecule to molecule or from chain segment to chain segment.

There are many factors at play when determining the rate of a charge-hopping process. For example, the molecules (or polymer chains) can have a distribution of level energies due to varying molecular conformations and micro-environment. It is then easier for a charge to hop from a higher-energy level to a lower-energy level on an adjacent molecule. Also, the presence of a charge leads to *intra*- as well as *inter*-molecular geometry relaxations, which depend on the nature of the electron-vibration couplings and determine the reorganization energy. When it comes to calculating the charge-transfer rates *k* from molecule *i* to molecule *j*, two formalisms are commonly used, which are based on Miller-Abrahams (MA) theory [37] and Marcus theory [38]:

(1) $$\begin{align*}{k_{\!M\!A}} =&\ {\nu_0}\exp \left( { - 2\gamma \left| {{R_{ij}}} \right|} \right)\nonumber\\  &\times\left\{{\begin{array}{@{}*{2}{c}@{}} {\exp \left({ - \frac{{{E_{\!j}} - {E_i}}}{{{k_B}T}}} \right)}&\quad {{E_{\!j}} > {E_i}}\\ 1&\quad {{E_{\!j}} \le {E_i}} \end{array}} \right.,\end{align*}$$

 

(2) \begin{equation*}{k_{\!M\!a\!r\!c\!u\!s}} = \frac{{2{\pi^2}{t^2}}}{{h\sqrt {\pi \lambda {k_B}T} }}\exp \left( - \frac{{{{\left( {{E_{\!j}} \!-\! {E_i} + \lambda } \right)}^2}}}{{4\lambda {k_B}T}}\right),\end{equation*}

where *v*_0_ denotes the attempted hopping frequency; *γ*, the inverse localization radius, which is a measure of the overlap of the wavefunctions on adjacent molecules; *R_ij_*, the distance from site *i* to *j; E_i_* and *E_j_*, the site energies; *T*, the temperature; *k_B_*, the Boltzmann constant; *h*, the Planck constant; *λ*, the reorganization energy; and *t*, the charge-transfer integral (electronic coupling). We note that there are some general considerations to keep in mind when applying these two formalisms [5]: the MA equation is usually better suited for the case of weak electron-vibration couplings (often found in rigid polymer segments) and low temperatures, while the Marcus formalism is better adapted to instances of strong electron-vibration couplings (often found in flexible molecular systems) and high temperatures.

## TRADITIONAL OFET DEVICE MODELS

The first OFETs were fabricated in the 1980s [39–41], with charge mobilities on the order of 10^−5^ cm^2^ V^−1^ s^−1^, some four orders of magnitude lower than that of amorphous silicon (0.5–1 cm^2^ V^−1^ s^−1^). Since then, great progress has been made regarding OFET performance. Modern OFET devices based on crystalline/well aligned materials now reach mobilities on the order of 10 cm^2^ V^−1^ s^−1^ or even higher [2,42].

**Figure 2.**
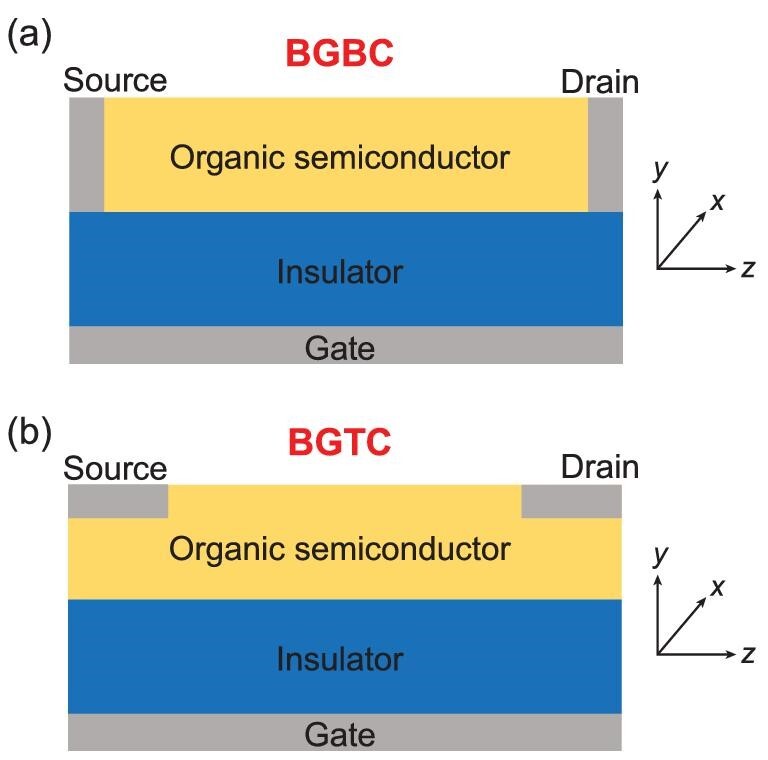
Illustration of the cross-sections (*yz*-plane) of (a) bottom-gate bottom-contact (BGBC) and (b) bottom-gate top-contact (BGTC) OFETs.

A first important aspect to recall is that OFET devices can have different architectures. Figure 2 illustrates two common configurations, the bottom-gate bottom-contact (BGBC) and bottom-gate top-contact (BGTC) configurations. In the former, the source and drain electrodes connect to the channel at the semiconductor–insulator interface, where most of the charge carriers are expected to transport; in the latter, the source and drain electrodes are away from the semiconductor–insulator interface, which means charge carriers need to travel across the OS film to reach the channel. Thus, these simple differences imply that the device configuration can influence the measured charge mobility.

**Figure 3.**
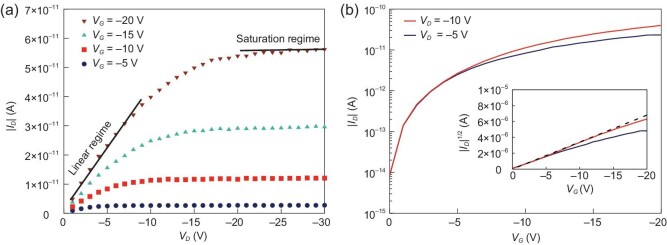
KMC-simulated (a) output and (b) transfer characteristics of a BGBC OFET device with a channel length of 1 μm. Solid lines in (a) highlight the linear and saturation regimes for *V_G_*= −20 V. Inset in (b) shows the evolution of the square root of the drain current as a function of gate voltage. The dielectric thickness is 16 nm, the relative permittivity is 4, and the channel width is 50 nm (adapted from ref. [15] with permission from Wiley-VCH).

We note that an OFET device has two operating regimes, a linear regime and a saturation regime. In the linear regime, the drain voltage (*V_D_*, the potential difference between the source and drain electrodes) is much smaller than the gate voltage (*V_G_*, the potential difference between the source and gate electrodes); here, increasing *V_D_* (while keeping *V_G_* fixed) leads to a higher current output (*I_D,lin_*), as illustrated in Fig. 3a. The saturation regime corresponds to instances where *V_D_* has become significantly higher than *V_G_*; in this regime, increasing *V_D_* (while keeping *V_G_* fixed) no longer increases the output current (*I_D,sat_*), see Fig. 3a. Understanding the details of the current characteristics in these two regimes can lead to a comprehensive description of OFET operation. However, gaining that understanding requires the use of an appropriate OFET device model.

This is where a major issue surfaces since, in spite of the substantial differences in the charge-transport mechanisms between organic and inorganic semiconductors, it turns out that the prevalent OFET device models were until recently directly borrowed from those originally developed for inorganic-based FETs. In these inorganic FET models, the currents are expressed as the following [43–45]:

(3) \begin{equation*}{I_{D,lin}} = \frac{{W{C_i}\mu }}{L}\left( {{V_G} - {V_T} - \frac{{{V_D}}}{2}} \right){V_D},\end{equation*}

 

(4) \begin{equation*}{I_{D,sat}} = \frac{{W{C_i}\mu }}{{2L}}{\left( {{V_G} - {V_T}} \right)^2}.\end{equation*}

Here, *W* is the channel width; *L*, the channel length; *C_i_*, the dielectric capacitance per unit area; and *V_T_* denotes the threshold voltage, which is the onset voltage for the current to flow.

Equations (3) and (4) correspond to the linear regime and saturation regime, respectively. While some model improvements were described [46–48], these two equations have remained the mainstream basis in the analysis of OFET devices [43,44]. We note that Equation (3) implies that the current is linear with applied gate voltage in the linear regime, while Equation (4) determines that the square root of current is linear with applied gate voltage in the saturation regime:

(5) \begin{equation*}\sqrt {{I_{D,sat}}} = \sqrt {\frac{{W{C_i}\mu }}{{2L}}} \left( {{V_G} - {V_T}} \right).\end{equation*}

These linear relationships are frequently used in the extraction of charge mobilities from OFET devices.

It is useful to look at some of the basic assumptions behind Equations (3) and (4) [45] and to discuss their relevance to organic semiconductors and OFETs:

- A first assumption is that the semiconductor is a continuum medium with uniform properties in space. The consequence for organic films is that the discrete molecular positions and variations in microscopic molecular levels are neglected. The trap densities and charge-transport properties are treated as position independent, while in reality they can vary across different layers.
- Factors such as disorder in the molecular packings and roughness of the dielectric–semiconductor interface are neglected. Thus, the organic molecules are assumed to be fully aligned along the substrate and the dielectric–semiconductor interface is taken to be perfectly flat.
- Ohmic contacts are assumed, which means that any contact resistance is not considered.

However, all these assumptions often do not hold true in the case of OSs [49], which has called for the development of more reliable and accurate OFET device models [49]. Optimally, OFET device models should include factors such as the presence of discrete molecular levels, disorder, anisotropy, traps, grain boundaries, complex film morphology and contact resistance. These factors are difficult to include as long as the organic semiconductor film is treated as a continuum medium. In other words, nano-scale, molecular-level details need to be incorporated into OFET device models. Needless to say, increasing resolution generally brings higher computational costs, which can limit the applicability of the models. Thus, the right approach has to combine the ability to consider a molecular resolution that can take full account of the specific features of charge transport in organic semiconductors, with the ability to maintain realistic computational costs. Such an approach is KMC, which has been widely exploited already in the modeling of organic light-emitting diodes and solar cells [12–14]. In the next section, we discuss the development of such a KMC OFET device model.

## DEVELOPMENT OF A KINETIC MONTE CARLO-BASED OFET DEVICE MODEL

**Figure 4.**
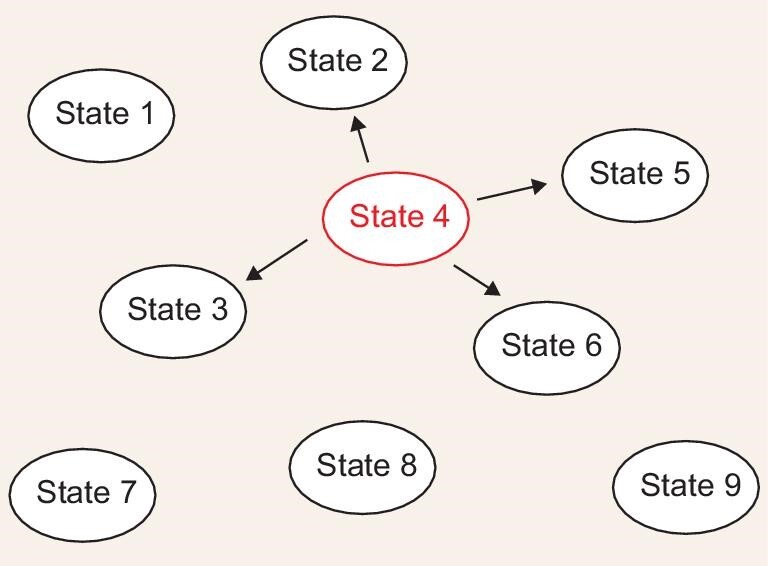
Illustration of the transition among states in the kinetic Monte Carlo algorithm. In this case, the system is in state 4 and can evolve to one of four states (2, 3, 5 or 6; we note that transition to the other states would not happen due to reasons such as the absence of any wavefunction overlap precluding electron transfer between state 4 and states 1, 7, 8 and 9). The state to which the system evolves is determined by Equation (6).

Kinetic Monte Carlo is an algorithm that describes the time evolution of a system [50]. The prerequisite for its application is that the system of interest can be represented by a set of discrete, distinguishable states (Fig. 4) and that the transitions among these states can be considered as uncorrelated. At any given time, the system can only be in one of these states. Provided that the transition rates from the current state to any other state can be evaluated, the next state the system can reach (denoted as *j*) satisfies:

(6) \begin{equation*}\frac{{\sum\limits_{i = 1}^{j - 1} {{k_i}} }}{{\sum {{k_i}} }} \lt {\xi _1} \le \frac{{\sum\limits_{i = 1}^j {{k_i}} }}{{\sum {{k_i}} }},\end{equation*}

where *k_i_* is the transition rate from the current state to any possible state *j* and *ξ_1_* is a random number uniformly distributed between 0 and 1.

The transition time *τ* is given by:

(7) \begin{equation*}\tau = - \frac{{\ln \left( {{\xi _2}} \right)}}{{\sum {{k_i}} }},\end{equation*}

where *ξ_2_* is also a random number uniformly distributed between 0 and 1.

KMC has been widely used in many research fields, encompassing chemistry, materials science, biology and physics [50]. When applying KMC to investigate a specific problem, the key aspect is how the states are defined. For example, when KMC is used to study charge transport in organic semiconductors [8,9], the molecules (or polymer segments) are simply taken as points, commonly referred to as sites, which a charge carrier can occupy; the ‘state’ then corresponds to a specific occupation of the charge carrier over these sites. The successful application of KMC to organic semiconductors has led to the widely used Gaussian Disorder Model (GDM) proposed in the 1990s, which provided a pivot microscopic description of charge transport in organic materials [9,10]. More recently, KMC has been used in the modeling of diode-structure devices [11], including organic solar cells [12,13] and light-emitting diodes [14].

A simulation of a device requires that multiple charge carriers be considered. The electrostatic interactions among charge carriers are then expected to exert a significant influence on their motions. Thus, an accurate evaluation of these interactions is critical to ensure reliable simulation results (we note that, hereafter, these electrostatic interactions are referred to as the external component of the site energy (*E_ex_*), as opposed to the internal component (*E_in_*) that corresponds to the molecular energy levels).

**Figure 5.**
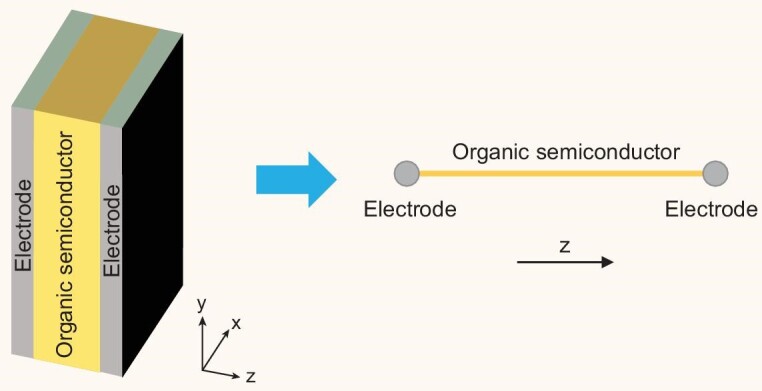
Illustration of a diode and its approximation in a 1D model.

Over the years, the one-dimensional (1D) Poisson equation has often been exploited to calculate the electric potentials across the diode, which were then used to evaluate *E_ex_* [12,51,52]. However, the 1D nature of this approach means that the device is treated as a 1D structure, say along the *z*-direction (see Fig. 5), with the carrier densities averaged along the *x*- and *y*-directions; as a result, short-range carrier–carrier repulsions/attractions on adjacent molecules (typically at distances on the order of 1 nm) are mostly neglected, which has actually been shown to have a detrimental impact on the quality of the charge-transport description [53]. One solution to this problem was a hybrid method proposed by van der Holst *et al*. [54]; there, the short-range interactions (at distances shorter than a preset cutoff) are directly calculated through Coulomb's law, while the long-range interactions (at distances longer than the cutoff) are evaluated by solving the 1D Poisson equation using layer-averaged carrier densities (while double counting in the spherical cutoff region is prevented). However, to properly describe the architecture of OFET devices, at least a two-dimensional (2D) approach is needed, as can be seen from Fig. 2.

When it comes to KMC modeling of OFET devices, only the linear regime (*V_D_* ≪ *V_G_*) can in practice be easily considered [55]. In this regime, the so-called gradual channel approximation (GCA) is generally applied. Considering the GCA implies that the electric potentials within the device can be approximated as the sum of two independent orthogonal vectors so that the horizontal (source-drain *z*-direction in Fig. 2) and vertical (*y*-direction) electric fields can be treated separately [55–57], which leads to a poor evaluation of the carrier–carrier electrostatic interactions. A more accurate modeling relies on using the Poisson equation but requires going beyond 1D and carrying out additional post-processing, as we describe below. Since in the case of OFETs the electric-potential variations most relevant to device operation occur in the *yz*-plane (see Fig. 2), the electrostatic interactions can in fact be calculated by solving the 2D Poisson equation:

(8) \begin{equation*}\frac{{{\partial ^2}\varphi }}{{\partial {y^2}}} + \frac{{{\partial ^2}\varphi }}{{\partial {z^2}}} = - \frac{\rho }{{{\varepsilon _r}{\varepsilon _0}}},\end{equation*}

where *ϕ* is the electric potential; *ρ*, the charge density; *ϵ_0_*, the vacuum permittivity; and *ϵ_r_*, the relative permittivity. Equation (8) has been originally applied by Wang *et al.* in their KMC modeling of OFETs in 2015 [58]; however, their simulations treated the OFET device as a strictly 2D system with carrier motions in the *x* direction (i.e. the channel-width direction) completely neglected, which is expected to lead to significant finite-size errors.

Another aspect that was not realized at first is that the straightforward application of the Poisson equation in KMC device modeling brings a carrier self-interaction error. Indeed, taking the electric potentials of the carrier population to calculate the external contribution to the site-energy difference,

(9) \begin{equation*}{E_{\!e\!x\!,j}} - {E_{\!e\!x\!,i}} = q\left( {{\varphi _j} - {\varphi _i}} \right),\end{equation*}

(where *q* is the charge of the carrier) includes the unphysical electrostatic interaction of the hopping carrier with itself (before and after the hopping event). This error has been shown to increase with the dimensionality of the Poisson equation [59]; in the case of the 2D Poisson equation, applying Equation (9) has been shown to lead to errors in the calculated currents that can be as large as 400%. To correct for this error, two algorithms have been proposed; they re-express Equation (9) either as [59]:

(10) \begin{equation*}{E_{\!e\!x\!,j}} - {E_{\!e\!x\!,i}} = q\left[ {\left( {{\varphi _j} - \varphi _j^{\left( i \right)} + \varphi _j^{\left( j \right)}} \right) - \left( {{\varphi _i}} \right)} \right],\end{equation*}

or as:

(11) \begin{equation*}{E_{\!e\!x\!,j}} - {E_{\!e\!x\!,i}} = q\left[ {\left( {{\varphi _j} - \varphi _j^{\left( i \right)}} \right) - \left( {{\varphi _i} - \varphi _i^{\left( i \right)}} \right)} \right].\end{equation*}

Here, *ϕ_j_^(i)^* denotes the electric potential at site *j* due to the presence of a single carrier at site *i*; it is obtained by solving the Poisson equation with the same boundary conditions but with all boundary values set to 0. Equation (10) can be understood as removing the hopping carrier from the system when calculating its hopping rates, while Equation (11) corresponds to moving the hopping carrier to the new position to obtain the correct electric potential after a hopping event. Importantly, since *ϕ_j_^(i)^* needs to be calculated only once and can be reused throughout the simulations as needed, these two algorithms do not significantly increase the computational cost.

On that basis, the electrostatic interactions among charge carriers in an OFET can be calculated by using the 2D Poisson equation with the carrier self-interaction error suppressed. We note that Equations (10) and (11) are also applicable to other types of organic electronic devices. In fact, they appear to be preferable over the hybrid approach mentioned above [59] since they eliminate the error due to the abrupt change in electric potential at the cutoff distance.

Another challenge when it comes to modeling OFET devices is that the channel length is often on the scale of micrometers. Decreasing this length in the simulations in order to reduce computational costs can alter the reliability of the results. Indeed, the ratio of channel length over film thickness needs to be maintained to preserve the OFET device characteristics; however, over-reducing the thickness affects the charge-transport properties within the conducting channel. To enable the modeling of large systems thus calls for the efficiency of the KMC device model to be greatly improved. It turns out that the most time-consuming parts in the KMC OFET simulations correspond to solving the Poisson equation and calculating the charge-transfer rates, both of which have to be repeated at every KMC step. It was then realized that, since each carrier hopping can be considered as removing one charge from position *j* and putting it at position *k*, the process of solving the Poisson equation can be replaced by addition operations [15]:

(12) \begin{equation*}{\varphi _i}\!\left( {n + 1} \right) = {\varphi _i}\!\left( n \right) - \varphi _i^{\left( j \right)} + \varphi _i^{\left( k \right)}.\end{equation*}

Importantly, all variables on the right-hand side of Equation (12) are known at every KMC step. Using such an algorithm then eliminates the need to invoke a Poisson solver at every KMC step, with the Poisson equation having to be solved once at the beginning of the simulation to obtain the initial values of the electric potential. As for the calculation of the charge-transfer rates, implementing a parallel-computing technique over all processors on a node has been demonstrated to achieve accelerations up to factors of ∼20 [15].

Recently, we were able to develop a KMC OFET device model that not only does not rely on the GCA but also works in both the linear and saturation regimes. This was accomplished by considering a Gaussian distribution (density of states) of the molecular-level energies in the organic semiconductors and hopping and injection rates based on the Miller-Abrahams expression. Figure 3 displays the calculated output and transfer characteristics of a BGBC OFET device with a channel length reaching a realistic value of 1 μm [15]. In that particular case, Ohmic contacts, a uniform organic film and a low disorder (51 meV, i.e. 2*k_B_T* at 298 K) were assumed. The figure illustrates that (i) the drain current first increases linearly with drain voltage and eventually saturates; (ii) the square root of *I_D_* is linear with the gate voltage for *V_G_* < *V_D_*; and (iii) the extrapolated threshold voltage is 0 V. All these features are consistent with those of actual OFETs [2,39,60], which points to the robustness of the KMC model we were able to develop [15]. Hereafter, we denote this model as the kinetic Monte Carlo OFET Model (KMCOM).

## APPLICATIONS OF THE KINETIC MONTE CARLO OFET MODEL

An appealing feature of the model we just described is that it is able to connect the microscopic charge-transport process to macroscopic device characteristics. This makes it an excellent platform to study OFET device physics and characteristics. In this section, we review some of the applications of KMCOM: We first deal with the properties of the conducting channel and the impact of the dielectric surface roughness, which are both fundamental aspects of OFET operation. Then we discuss the nonlinear OFET characteristics that have been encountered in a number of instances.

### Effective channel thickness

In an OFET, charge carriers do not transport through the entire organic film. Instead, they are expected to mainly reside in a region close to the dielectric that has been polarized by the gate voltage [61]. The characteristics of this effective channel including its thickness are thus a fundamental aspect of OFET operation. A usual assumption, referred to as the charge-sheet model, assumes a zero effective channel thickness, which means that all charge carriers would exclusively transport within the organic layer adjacent to the dielectric. This assumption, however, is generally not valid for OFETs since investigations into the effective channel thickness have shown that the drain currents and mobilities in OFET devices saturate around 3–10 monolayers [62–65]. Also, numerical analyses based on the GCA have indicated that charge carriers can distribute over several organic layers as a function of applied gate voltage [61].

**Figure 6.**
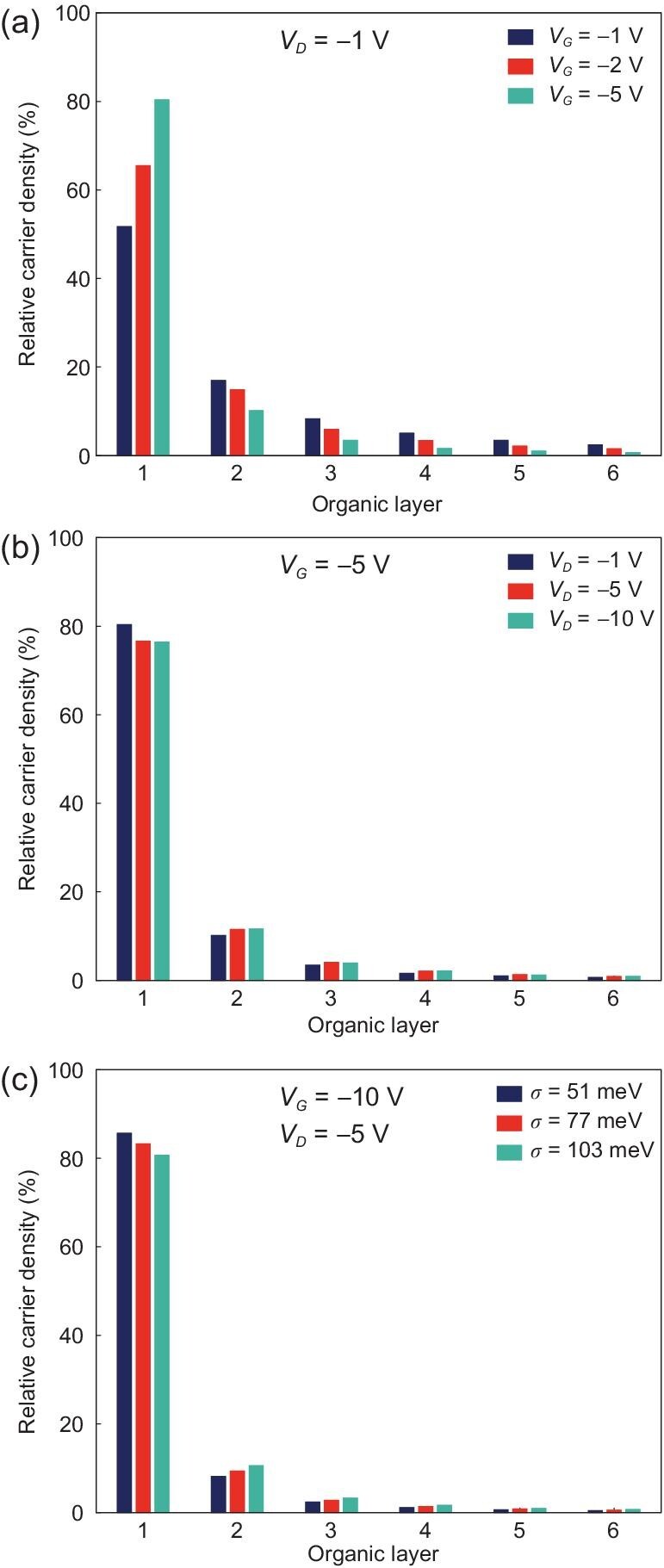
KMCOM-simulated (a) relative carrier density in different organic layers (labeled when starting from the organic semiconductor/dielectric interface) in an OFET device as a function of gate voltage at *V_D_* = −1 V; (b) relative carrier density as a function of drain voltage at *V_G_* = −5 V; (c) relative carrier density as a function of energetic disorder *σ* at *V_G_* = −10 V, *V_D_* = −5 V. The relative permittivity is 4 and the dielectric thickness is 16 nm, which is about 1/20 that of common OFET devices fabricated in labs; *V_G_* used in the simulations thus correspond to ∼20 times larger values when relating to those devices. In (a) and (b), the energetic disorder is 51 meV (adapted from ref. [15] with permission from Wiley-VCH).

Here as well, our KMCOM proved to be useful. While the simulations point to most of the carriers moving within the first organic layer over the dielectric (e.g. when considering a dielectric thickness of 16 nm and a relative permittivity of 4, this layer holds about 50% of the carriers at *V_G_*= −1 V), the carrier distributions in the next layers are far from being negligible, as illustrated in Fig. 6. The effective channel thickness ranges from ∼5 nm to over 10 nm, beyond which the layers have relative carrier densities lower than 1%; this result is consistent with experimental observations [62–65]. The main message here is that it is indeed inappropriate to neglect the channel thickness, as is routinely done by applying the charge-sheet model to OFETs. In addition, the effective channel thickness is not constant but rather influenced by the magnitudes of the gate voltage, drain voltage and energetic disorder *σ* in the organic semiconductor:

- Increasing the gate voltage leads to decreased effective channel thickness, in agreement with earlier numerical simulations [61].
- Increasing the drain voltage slightly reduces the relative carrier density in the organic layer in contact with the dielectric, which results in thicker effective channel thickness. However, when the saturation regime is reached, a further increase in the drain voltage has no effect.
- The presence of a higher extent of energetic disorder reduces the number of carriers in the first organic layer and leads to an increased effective channel thickness. Indeed, the carriers trapped in low-energy sites weaken the impact of the vertical electric field (perpendicular to the organic-dielectric interface) that confines carriers near the interface.

These results illustrate that, in order to ensure reliable results, the thickness of the conducting channel needs to be considered at large *V_D_*/*V_G_* ratios and/or for organic semiconductors with a high degree of disorder.

### Dielectric surface morphology

Since the majority of charge carriers are close to the dielectric, the characteristics of the dielectric-organic semiconductor interface naturally impact OFET performance. Early OFET device models often assumed a perfectly flat interface, which turns out to be a very crude approximation. Indeed, it has been shown experimentally that modifying the dielectric surface roughness can lead to significantly different device characteristics [66–70]. While it is difficult to describe a rough dielectric surface via an analytical approach, the inclusion of rougher dielectric surfaces is straightforward within KMCOM. In this section, we discuss its application to non-flat dielectric surfaces.

**Figure 7.**
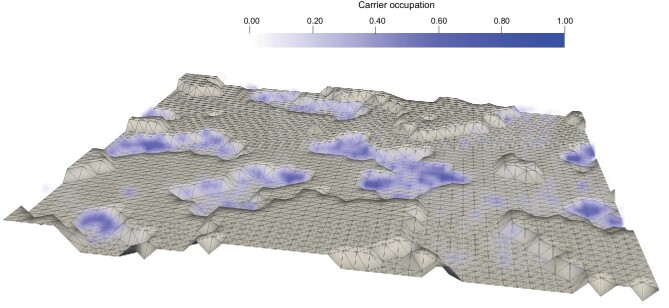
KMCOM-simulated averaged 3D carrier occupation overlapped with a simulated dielectric surface morphology with a lateral size of 50 nm × 50 nm (along the *xz*-directions) inside an OFET device. The gate voltage and drain voltage are −10 V and −5 V, respectively; the dielectric thickness is set at 16 nm; the relative permittivity, at 4; and the energetic disorder, at 51 meV. The dielectric surface is marked in gray with grids. The carrier occupational probabilities, which are represented in blue, are essentially within the ‘valleys’ (adapted from ref. [71] with permission from Wiley-VCH).

**Figure 8.**
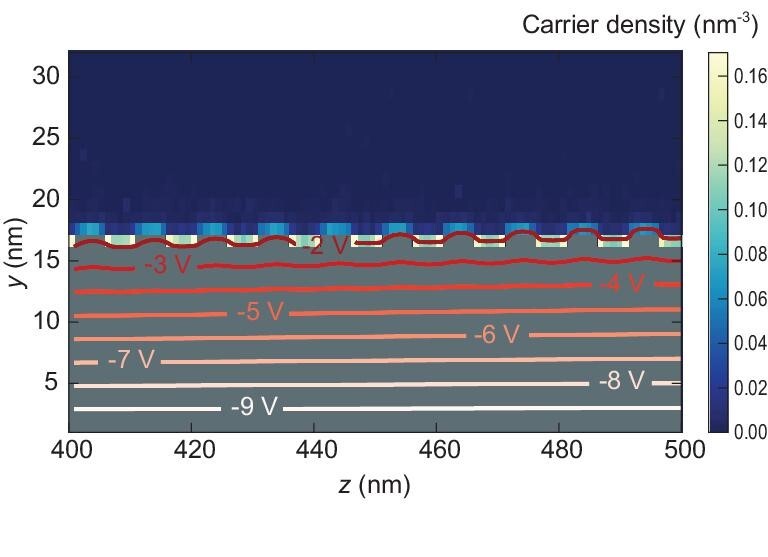
KMCOM-simulated carrier densities in the *yz* plane for a morphology with microgrooves along the channel width direction (where *z* defines the source-to-drain direction and *y* is perpendicular to the semiconductor-dielectric interface). The color bar represents the carrier density (in nm^−3^). The isolines describe the electric potential in the *yz* plane. Note that the variations in electric potential along the vertical direction above the semiconductor-dielectric interface are very small. The dielectric is represented in gray. The gate voltage and drain voltage are −10 V and −5 V, respectively; the dielectric thickness is set at 16 nm; the relative permittivity, at 4; and the energetic disorder, at 51 meV (adapted from ref. [71] with permission from Wiley-VCH).

Figure 7 illustrates the surface geometry of a specific simulated dielectric [71] and the KMCOM-simulated carrier occupations. Since the electric field in the device is mostly aligned normal to the interface, most carriers are present in ‘valleys’ while avoiding ‘hills’. This effect is also seen in Fig. 8, which models a dielectric surface with microgrooves present along the channel-width direction. This geometry-dependent carrier distribution significantly impacts device performance:

- The ‘valleys’ act as shallow traps; charge carriers in these ‘valleys’ have to climb out in order to transport in the channel. The result is a reduced effective carrier density, which in turn leads to lower overall current output.
- ‘Hills’ reduce the areas available for charge transport and lead to longer charge-transport pathways; the result is a lower effective charge mobility.

Both of these effects are detrimental to OFET performance. For instance, we modeled a series of dielectric surface morphologies with roughness ranging from 0 to 2 nm; the results indicate variations in charge mobility *μ* by over one order of magnitude. Interestingly, log*μ* decreases approximately linearly with the dielectric surface roughness. These trends are both consistent with available experimental data [72–79]. However, a rough dielectric surface does not necessarily always lead to reduced charge transport. Our KMC simulations have shown that microgrooves along the channel length direction have little influence on the overall charge-transport efficiency, although the charge carriers preferably distribute in these grooves [71].

### Nonlinear current characteristics

**Figure 9.**
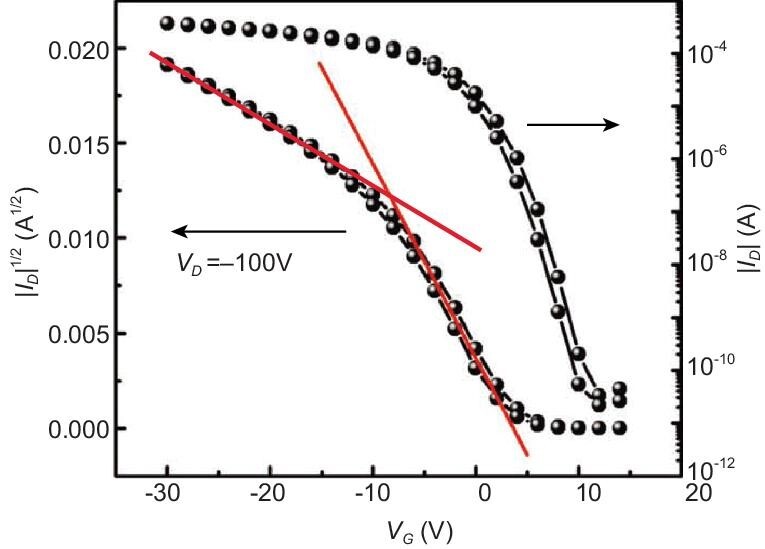
Illustration of nonlinear current characteristics in an OFET device. The red lines highlight the low-voltage and high-voltage regions (adapted from ref. [80] with permission from Wiley-VCH).

As discussed in the `Traditional OFET device models' section, in the linear regime, the current is predicted to evolve linearly with gate voltage while in the saturation regime it is the square root of the current that is predicted to have a linear evolution. These relationships form the basis for the determination of the charge mobility of OSs in an OFET configuration. However, it has been found that such linear relationships can be violated [49]. In particular, there can occur two regions: a low-voltage region with a larger slope and a high-voltage region with a smaller slope, as illustrated in Fig. 9. This has raised an obvious question: which region should be used to calculate the charge mobility [49]? A common choice has been to choose the low-voltage region, for the simple reason that it gives higher charge mobilities (e.g. values as high as 43 cm^2^ V^−1^ s^−1^ have been reported for 2,7-dioctyl[1]benzothieno[3,2-b][1]benzothiophene (C8-BTBT) [81]). However, is this necessarily the right approach?

Several possible origins of these nonlinear current characteristics have been discussed, including the presence of a gate-voltage dependent contact resistance [82,83] and/or charge trapping [84]. Consideration of these mechanisms suggests using the high-voltage region to calculate the charge mobility, since the low-voltage region is impacted by factors beyond the charge-transport characteristics. With that in mind, the charge mobility measurements based on OFET devices have been recently re-examined, with most mobilities initially reported to be larger than 10 cm^2^ V^−1^ s^−1^ considered to be likely unreliable [85]. In order to improve consistency in the mobilities measured across different research groups, guidelines have been provided to calculate the charge mobility in the presence of nonlinear currents [44,86]. However, our understanding of the nonlinear characteristics remains incomplete as not all nonlinearities can be explained by the mechanisms proposed early on. This has become a serious issue in OFET-related studies, as highlighted in articles dedicated to this topic, see e.g. reviews by Nguyen [87], Pei [88] and their co-workers.

**Figure 10.**
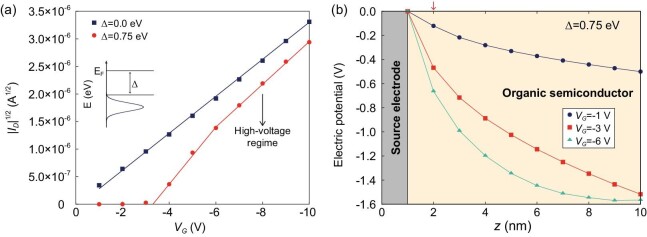
(a) KMCOM-simulated *I_D_*^1/2^ ∼ *V_G_* curve in the presence of an injection barrier (illustrated in the inset). (b) Electric potentials in the first organic layer from the semiconductor-dielectric interface at different positions along the *z* direction. The drain voltage is -10 V in all cases; the dielectric thickness is set at 16 nm; the relative permittivity, at 4; and the energetic disorder, at 51 meV. The red arrow marks the organic site that is in direct contact with the source electrode (adapted from ref. [71] with permission from Wiley-VCH).

KMCOM has also proven useful in the understanding of OFET nonlinear characteristics, in spite of the fact that it is currently based entirely on the hopping mechanism. The KMCOM results confirm the experimental data describing how contact resistance leads to nonlinear currents (Fig. 10a) and provide additional microscopic insights. The appearance of a non-Ohmic metal-organic semiconductor contact can be modeled by explicitly introducing an injection barrier (Δ) in the calculation of the site energy difference between the electrode sites and the OS sites. Increasing the gate voltage lowers the electric potential at the organic sites adjacent to the source electrode (Fig. 10b); this, in turn, results in lower effective injection barriers and exponentially increased injection rates (see Equation (1)). Therefore, the contact resistance at the electrode-organic semiconductor interface becomes gate-voltage dependent. This effect is prominent when there is a larger mismatch between the work function of the metal and the ionization energy or electron affinity of the organic semiconductor. As a result, nonlinearities occur with the *I_D_*^1/2^-*V_G_* curve bending downward compared to the traditional linear relationship: the low-voltage region has a larger slope while the high-voltage region has a smaller slope. These results from KMCOM, which describe the device characteristics entirely based on microscopic processes, are consistent with previous experimental findings based on pentacene, rubrene and 2,9-didecyl-dinaphtho[2,3-b:2',3'-f]thieno[3,2-b]-thiophene (C_10_DNTT) [82,83,89]. In this situation, the low-voltage region is significantly affected by the impact of the gate voltage on the contact resistance and thus does not genuinely reflect the charge-transport properties of the organic semiconductor. As a result, in such a situation, the high-voltage region should be used to evaluate the charge mobilities. However, our simulations show that extracting mobilities based on the high-voltage region can still lead to up to ∼25% overestimated values compared to devices with no contact resistance. Therefore, the calculation of charge mobilities from nonlinear current characteristics should continue to be treated with much care.

**Figure 11.**
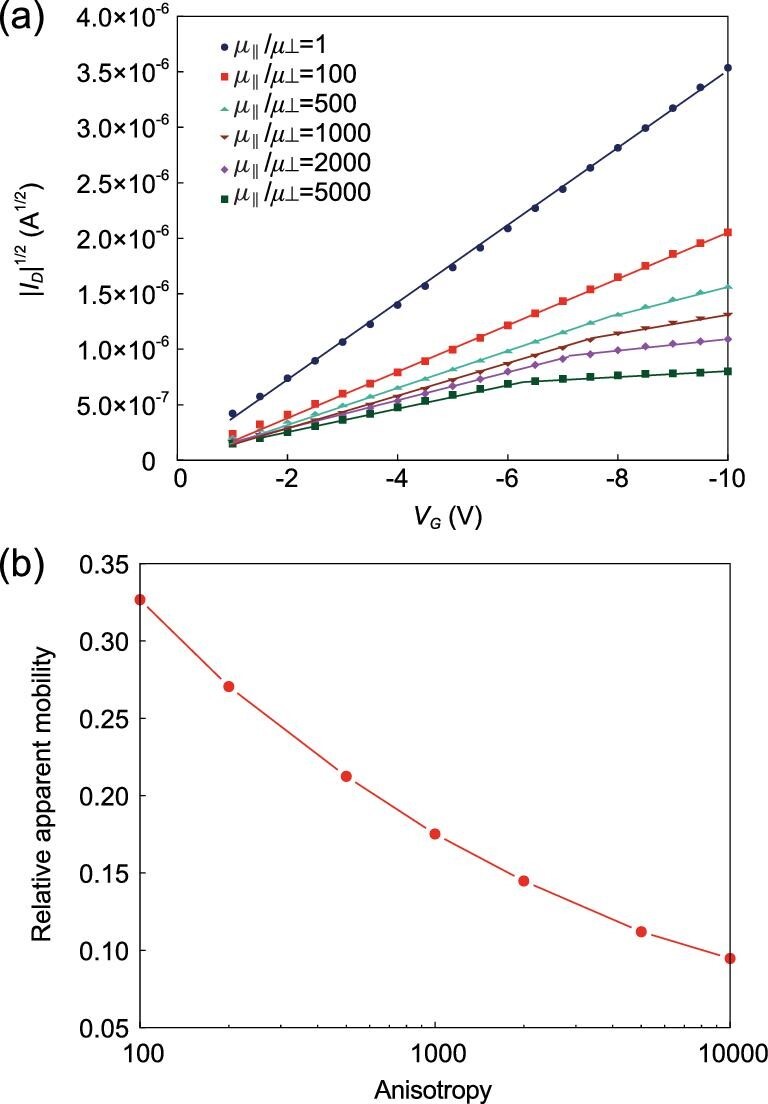
(a) KMCOM-simulated *I_D_*^1/^^2^ vs *V_G_* curve in the case of quasi-1D charge transport in a BGTC OFET device. The drain voltage is set at −10 V; the organic semiconductor film thickness and the dielectric thickness are both 16 nm; the relative permittivity is 4. (b) Relative apparent mobility in the channel direction as a function of charge-transport anisotropy (defined as *μ_//_/μ*_⊥_), calculated from the low-gate voltage regime in (a) (adapted from ref. [95] with permission from the American Chemical Society).

Simulations based on KMCOM have also revealed another possible origin for the nonlinear current characteristics. Often, the deposition of organic semiconductor is carried out to ensure that the organic film is highly aligned. In particular, polymer chains are made to orient along the channel direction [90,91] and/or highly crystalline molecular materials [27,92] are used. Since charge transport in organic semiconductors is highly anisotropic (e.g. charge mobilities along polymer chains can typically be some three orders of magnitude higher than inter-chain mobilities [93,94]), charge transport in such OFET devices can have a quasi-1D nature. When using KMCOM to simulate the device characteristics in such cases, the simulations indicate that the quasi-1D nature of transport can indeed lead to nonlinear currents when current injection takes place away from the channel (which can occur in a BGTC configuration or in the case of poor contacts near the channel), as shown in Fig. 11a [95]. In this instance, charge transport occurs in the bulk of the organic film; the gradual channel approximation and the charge-sheet model are then invalid. The degree of nonlinearity depends on the charge-transport anisotropy as well as the thickness of the OS film. When this mechanism applies, the charge mobilities evaluated from the low-voltage region are underestimated (Fig. 11b); in contrast to the case of contact resistance, the high-voltage regime should be used here to calculate the charge mobilities.

## CONCLUSION AND OUTLOOK

In many instances, organic semiconductor films have disordered, complex morphologies that result in a transport mechanism dominated by hopping. To take full account of these features, OFET device models need to provide for molecular-level resolution. KMC is a theoretical approach that allows the realization of such molecular-level OFET models. These models have significantly improved over the past few years and are now capable of describing micrometer-long devices that are comparable to real devices and of evaluating the device characteristics in both the linear and saturation regimes.

The KMC OFET device model (KMCOM) we developed has been successfully applied to address some of the fundamental issues relevant to OFET devices, such as the effective thickness of the channel and the role of the dielectric surface morphology. We note that KMCOM is currently based on the hopping mechanism of charge transport, which makes it suitable for devices using amorphous organic semiconductor films or crystals with weak intermolecular electronic couplings. In spite of this limitation, it has also been shown to be a useful tool in the understanding of the nonlinear features that can appear in high-mobility OFETs, although an explicit consideration of the delocalization aspects is yet to be incorporated into the model. To achieve better accuracy and wider applicability, further developments are required. In particular, additional studies need to address the following points:

- *Explicit inclusion of the details (at the nano-scale) of the morphology of the organic-semiconductor film*. This aspect has not been thoroughly incorporated due to the lack of experimental and theoretical tools that can probe the morphological details at the molecular level. While a KMC OFET model can, in principle, integrate a complex film morphology, to achieve this goal still requires improvements in the description of the electrostatic interactions and in computational efficiency, as described below.
- *Improvements in the description of the electrostatic interactions*. Since many charge carriers are present simultaneously in an OFET device (a typical carrier density in the conducting channel is up to ∼10^20^ cm^−3^ [96]), more accurate evaluations of their electrostatic interactions are called for to ensure reliable simulation results. While KMCOM relies on the 2D Poisson equation, in principle, higher accuracy can come from exploiting the 3D Poisson equation when realistic film morphologies are considered. However, this comes with a much higher computational cost.
- *Improvement in computational efficiency*. It is of crucial importance to optimize and accelerate the KMC OFET device model in order to broaden its applicability. One potential solution is to rely on parallel computing, a point that has been discussed briefly in the `Development of a kinetic Monte Carlo-based OFET device model' section. Also, it would be useful to further explore master equation simulations [97], as these have been shown to be less demanding than KMC; another potential advantage of master equation simulations is that, since the carrier motions proceed simultaneously in such simulations, they can be easily accelerated via the use of GPUs [96,98].
- *Impact of delocalization effects*. While the present KMC OFET models are based on the hopping charge-transport mechanism, as discussed in the `Charge transport in organic semiconductors' section, band transport and charge-delocalized regimes become prevalent in high-mobility organic semiconductors. Incorporating delocalization into KMC modeling is possible [99] and can help gain a more comprehensive understanding of OFET device physics.

We envision that through such continuous developments, molecular-level OFET device models will become an increasingly useful platform in the investigation of OFET devices and serve as a complementary tool for routine data analysis.
